# Molecular progression to cervical precancer, epigenetic switch or sequential model?

**DOI:** 10.1002/ijc.31549

**Published:** 2018-07-03

**Authors:** Belinda Nedjai, Caroline Reuter, Amar Ahmad, Rawinder Banwait, Rhian Warman, James Carton, Sabrina Boer, Jack Cuzick, Attila T. Lorincz

**Affiliations:** ^1^ Barts and the London School of Medicine, Charterhouse Square Centre for Cancer Prevention, Wolfson Institute of Preventive Medicine London EC1M 6BQ United Kindom; ^2^ Department of Histopathology Charing Cross Hospital, Fulham Palace Road London W6 8RF United Kingdom; ^3^ Department of Urology Radboud University Medical Center, Radboud Institute for Molecular Life Sciences Nijmegen The Netherlands

**Keywords:** methylation, epigenotype, human papillomavirus, biomarker, cervical cancer, cervical intraepithelial neoplasia (CIN), HPV typing, molecular switch, sequential progression

## Abstract

The evolution of precancerous cervical lesions is poorly understood. A widely held model of cervical intraepithelial neoplasia grade 3 (CIN3) development is sequential progression from normal through CIN1 and CIN2 to CIN3. Another hypothesis, the “molecular switch” model, postulates that CIN3 can evolve directly from human papillomavirus (HPV)‐infected normal epithelium without progressing through CIN1 and CIN2. To shed light on this process, we compared DNA methylation of selected human biomarkers and HPV types in two groups of CIN1: CIN1 that were near or adjacent to CIN3 (adjacent‐CIN1) and CIN1 that were the principal lesions with no CIN3 detected (principal‐CIN1). 354 CIN (CIN1 and CIN3) and normal tissue areas were dissected and typed for HPV from 127 women who underwent loop electrosurgical excision procedures (LEEP). Methylation of genes *EPB41L3* and the viral regions of HPV16‐L1/L2, HPV18‐L2, HPV31‐L1, and HPV33‐L2 were determined by a highly accurate quantitative pyrosequencing of bisulfite converted DNA. There was a significant trend of increased methylation with disease grade comparing normal to CIN1 and CIN3 (*p* < 0.0001). Adjacent‐CIN1 predominantly shared the same HPV types as the CIN3, however, methylation differed substantially between adjacent‐CIN1 and CIN3 (*p* = 0.008). In contrast diagnostically principal‐CIN1 had an indistinguishable methylation distribution compared to adjacent‐CIN1 (*EPB41L3: p* = 0.49; HPVme‐All: *p* = 0.11). Our results suggest that progression from normal epithelium to CIN1 or CIN3 is usually promoted by the same HPV type but occurs via distinct DNA epigenotypes, thus favoring the “molecular switch” model.

## Abstract 2

**What's new?**

Do cervical intraepithelial neoplasias always start at CIN1 and progress to CIN3? Or can CIN3 arise directly from normal epithelium, after HPV infection? The authors investigated by testing DNA methylation in different CINs. They compared CIN1 with CIN3 within the same cervix, and also compared CIN1 adjacent to CIN3 with CIN1 from patients without detectable CIN3. CIN1 and their neighboring CIN3, they found, each had distinct patterns of methylation, despite carrying the same HPV infection. All the CIN1 samples, however, exhibited similar methylation, regardless of the presence of CIN3. This suggests methylation can switch cells directly into a CIN3 state.

**Abbreviations**
Adj: adjacent
AVER: average
CIN: cervical intraepithelial neoplasia
CpG: 5'‐cytosine‐phosphate‐guanine‐3'
CTT χ^2^: Cuzick test for trend statistic
DNA: deoxyribonucleic acid
hr: high‐risk
E2BS: E2 binding site
EDTA: ethylenediaminetetraacetic acid
FFPE: formalin‐fixed paraffin‐embedded
HCl: hydrochloric acid
HPV: human papillomavirus
HPV16: human papillomavirus type 16
HPV18: human papillomavirus type 18
HPV31: human papillomavirus type 31
HPV33: human papillomavirus type 33
HPVme: HPV methylation
IMP: data imputation
KWT χ^2^: Kruskal‐Wallis test statistic
LEEP: loop electrosurgical excision procedure
n: number of samples
Norm: normal
qPCR: quantitative polymerase chain reaction
Prin: principal
SDS: sodium dodecyl sulphate
TSS: transcription start site
URR: upstream regulatory region.

## Introduction

**Figure 1:**
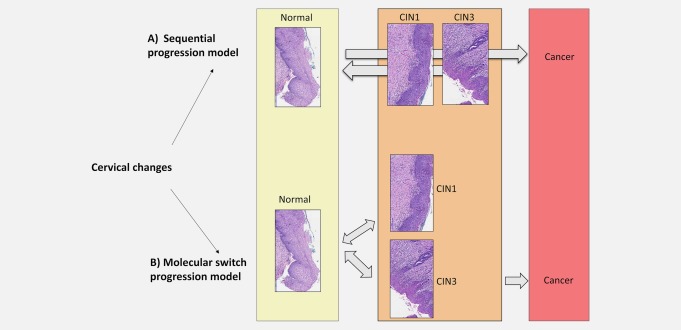
Current alternative cervical cancer progression models. In the sequential progression model (*A*), a long‐lasting HPV infection causes cervical intraepithelial neoplasia (CIN) changes in consecutive steps from low grade CIN1 to high grade CIN3 and finally cancer. An alternative concept, the molecular switch model (*B*), assumes that CIN1 is not necessary for the development of CIN3. CIN3 could evolve straight from normal epithelium infected by HPV. Here, we propose that distinct epigenotypes trigger distinct morphologic changes such as CIN1 or CIN3 independently, explaining partially the versatile nature of these lesions. Double‐sided arrows indicate possible regression.

Human papillomavirus (HPV) infections account for an estimated 530,000 new cervical cancers and 270,000 deaths annually, most of which occur in developing countries.^1^, ^2^, ^3^ >25 types of HPV are transmitted through sexual contact but most infections do not cause symptoms and are cleared after a short time. When an infection is persistent with one or more high‐risk HPV (hrHPV) there is an increased chance of cervical intraepithelial neoplasia (CIN2 or CIN3).^4^, ^5^, ^6^, ^7^, ^8^ It was originally thought that cervical cancer evolved from HPV infected normal cervical epithelium via a “sequential progression” model (Fig. 1). In this model, a long‐lasting HPV infection would cause cervical intraepithelial changes in a consecutive way, from HPV infected normal tissue to CIN1, CIN2, CIN3 and finally cancer.^9^ However, an alternative idea is that CIN1 may not be necessary for the development of CIN3^10^, ^11^ and that CIN3 could evolve directly from normal epithelium infected by HPV after a “molecular switch” model (Fig. 1). This means clinically relevant CIN3 may develop fairly rapidly after HPV infection.^12^ As a consequence, CIN1 lesions in general and most CIN2 may not be precursor stages of cervical cancer, but rather the visible effects of a productive HPV infection. It may then take 10–12 years to develop invasive cervical cancer from CIN3.^13^, ^14^ A clearer understanding of molecular changes leading to CIN3 could provide more accurate biomarkers to improve diagnosis and therapy.

Looking at the methylation level of certain genes is a way to shed light on the molecular progression of precancerous cervical lesions. DNA methylation is a reproducible physical epigenetic change involved in a variety of cellular processes that plays an important role in cancer progression.^15^ Numerous studies have highlighted the importance of host and viral gene methylation in the development of cervical cancer and potential use as biomarkers to triage HPV positive patients.^16^, ^17^, ^18^, ^19^, ^20^, ^21^, ^22^, ^23^, ^24^ A strong association has been observed between DNA methylation patterns and CIN of various grades in both cervical scrapes and biopsies. Typically, higher methylation is observed in patients with advanced intraepithelial lesions and carcinoma.

**Figure 2:**
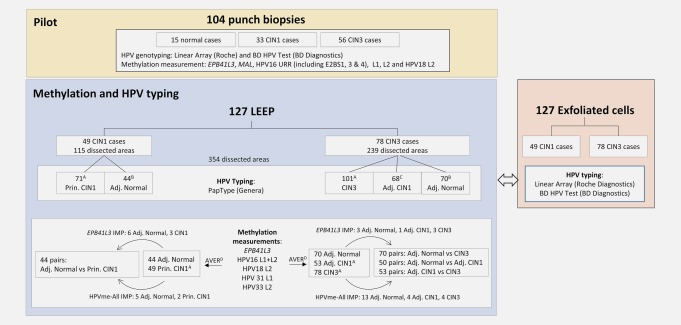
Flow chart showing the number of cases and dissected areas used for the main study including loop electrosurgical excision procedure (LEEP; tissues, centre main panel) and punch biopsies for the pilot study (top main panel). For some cases several CIN1 or CIN3 areas were dissected per cervix (*A*). Sections of five CIN1 and eight CIN3 cases had no normal epithelium (*B*). Sections of ten CIN3 cases had no adjacent‐CIN1 lesions (*C*). Mean methylation levels were averaged per lesion type (*D*). HPV genotyping was done separately for all lesions. Our study also included a comparison of HPV typing data from the 127 LEEP to corresponding exfoliated cell specimens (right panel). Abbreviations; Adj., adjacent; AVER, data averaged per lesion type; CIN, cervical intraepithelial neoplasia; IMP, data imputation; Prin., principal. [Color figure can be viewed at http://wileyonlinelibrary.com]

In our study, we investigated whether methylation data could bring evidence in support of either the molecular switch or the sequential progression model or both. We used epigenetic markers and HPV typing to investigate the two main models of precancerous cervical disease progression in areas of cervical lesions with discrete coexisting foci of different grades. We sought to determine whether levels of DNA methylation were a characteristic of lesion grade. Our primary aims were to contrast methylation in CIN1 in two possible configurations: (*i*) adjacent‐CIN1 versus CIN3 from the same women and (*ii*) adjacent‐CIN1 versus principal‐CIN1 lesion from different women. We hypothesised that if methylation levels were different between adjacent‐CIN1 and CIN3 on the same cervix but similar between adjeacent‐CIN1 and principal‐CIN1, this would add support to the “molecular switch” model. On the other hand, if the methylation levels overlapped substantially between grades and adjacent‐CIN1 exhibited higher methylation levels than principal‐CIN1 then; “the sequential progression” model would be favored. A secondary aim was to determine whether adjacent lesions were usually infected by the same or different HPV types. The study was made possible by our collection of carefully annotated Loop Electrosurgical Excision Procedure (LEEP) cervical tissues (Fig. 2).

## Material and Methods

Our goal was to compare HPV types and DNA methylation patterns in different kinds of cervical lesions that we obtained from the LEEP surgical tissues. Tissue areas to dissect were identified by microscope‐based pathology review and annotation.

### Study population and clinical specimens

Archived material from the Predictors studies (Ethics no. 05/Q0406/57) collected from 2005 to 2009 was used.^25^, ^26^ All patients with moderate and higher dyskaryosis in the baseline liquid‐based cytology and those with persistent mild abnormalities were sent for colposcopy. Before colposcopic examination an exfoliated cell specimen was taken from the cervix into PreservCyt (Hologic Inc., MA, USA) for HPV typing. Additionally, punch biopsies were taken from areas with abnormal appearance to determine the histological diagnosis of the patients. Final histopathological diagnoses were based on reviews by at least two pathologists. The highest grade of abnormality seen in the biopsy was used. If the pathologist panel review confirmed CIN2 or worse (CIN2+), patients underwent treatment by LEEP and the surgical tissues were fixed in formalin and embedded in paraffin (FFPE). We used FFPE punch biopsies from 104 women and FFPE LEEP tissues from 127 women (Fig. 2). We excluded women who had a final diagnosis of CIN2 from the study to minimize confusion from inaccurate pathology diagnoses; this was because of strong evidence that CIN2 in particular is a less reliable diagnosis than CIN1 or CIN3 and may not be a distinct biological entity.^27^, ^28^, ^29^, ^30^, ^31^

### Lesion dissection, annotation and DNA extraction

For the biopsies, twelve 5 μm FFPE sections of normal, CIN1 and CIN3 were cut on a microtome using a new blade for each block. Sections were stored at −70°C until DNA extraction. Entire sections were scraped from the slides using a scalpel and deparaffinised using three washes in xylene and one wash with 100% ethanol. DNA was extracted using QIAamp DNA FFPE Tissue Kit (Qiagen, Hilden, Germany) according to manufacturer's instructions with an initial incubation step at 56°C for 16–18 hr with proteinase K. The DNA concentration was measured using a NanoDrop 1000 Spectrophotometer (Thermo Fisher Scientific, Waltham, MA).

For the LEEP blocks, sections were cut using a sandwich cutting procedure^32^ using a new blade for each block (Supporting Information Fig. S1).^32^ Negative controls (breast tissue) were used after every 10 blocks to rule out contamination. The negative controls all tested negative for HPV. Twelve 5 µm FFPE sections of the 127 LEEP specimens were cut. The first and last sections were H&E stained and the remaining ten sections were used for DNA extraction. The H&E slides were carefully marked by an expert histopathologist (J Carton) to indicate different areas of CIN and normal tissues and used as guides for subsequent precise scalpel‐based dissection of the corresponding unstained sections under low power magnification. Sections were separated into two categories (CIN1 and CIN3 cases) depending on the highest‐grade lesion found. Where possible, normal squamous epithelium adjacent to CIN1 were also dissected. The CIN3 cases also included additional tissue (where possible) extracted from adjacent normal and CIN1 areas. Only one (if any) adjacent normal area was dissected per CIN1 or CIN3 case. However, if sections contained multifocal CIN1 or CIN3 lesions, additional areas were dissected and processed separately. Scraped areas were separated into different tubes for DNA extraction. Because of the small amount of starting material, dissected areas were deparaffinized using 160 µl of hexadecane followed by a 5‐min incubation at 56°C. Two hundred microliters of universal extraction buffer containing 50 mM Tris‐HCl pH 8.0, 1 mM EDTA and 0.05% SDS was added to tissues along with 400 mg of Proteinase K (Qiagen) and incubated overnight at 56°C followed by a 1‐hour incubation at 90°C. The lower phase was then transferred to a new tube and stored at −20°C before PCR.

### HPV type detection

The 354 dissected areas from LEEP samples were tested using the PapType High Risk HPV Detection and Genotyping kit (PapType kit, Genera Biosystems Ltd, Victoria, Australia) according to the manufacturer's instructions (Fig. 2). The kit is able to detect 13 hrHPV types (HPV16, 18, 31, 33, 35, 39, 45, 51, 52, 56, 58, 59, and 68), one possibly hrHPV type (HPV66) and two low‐risk types (HPV6 and 11). The PapType test was performed with 10 µl of DNA in a final reaction volume of 20 µl with the addition of 0.4 µl of Tween 20 (2%). The PCR reaction amplifies a variable region of the L1 gene. A fragment of the human cardiac myosin light chain gene (MLC‐1) was co‐amplified in the same reaction vessel as a quality and quantity control. The results of HPV typing from the LEEP samples were compared to each other and their corresponding cervical scrape samples taken before colposcopy (Fig. 2). The exfoliated cells were genotyped by Linear Array (Roche Diagnostics, Rotkreuz, Switzerland) for Predictors 1 and by BD HPV test (BD Diagnostics, Franklin Lakes, NJ) for Predictors 2.^25^, ^26^

### DNA methylation assays

DNA methylation was measured by a highly accurate pyrosequencing assay as previously described.^33^ Sodium bisulfite conversion of the genomic DNA was done using 200 ng DNA obtained from the punch biopsies and 20 μL of the DNA extract from the LEEP sections using the EZ DNA Methylation Kit (Zymo Research, Irvine, CA) following the manufacturer's instructions.

The punch biopsies were used to determine the most informative HPV regions for the main study using the LEEP sections (Fig. 2). Analysed human regions on the biopsies were based on our and others' previous work and included *EPB41L3* (CpG sites 425, 427, and 438 relative to transcription start site [TSS]) and *MAL* (CpG sites 529, 533, 535, 539, and 542 relative to TSS).^24^, ^34^ Viral regions included the HPV16‐URR (CpG sites 31, 37, 43, 52, 58, 7428, 7434, 7455 and 7461, which comprises E2BS1, 3 and 4), HPV16‐L1 (CpG sites 6367 and 6389), HPV16‐L2 (CpG sites 4238, 4247, 4259, 4268, 4275) and HPV18‐L2 (CpG sites 4256, 4261. 4266, 4269, 4275, 4282).^21^, ^22^ None of the samples were excluded on the basis of their HPV type. Amplification of CpG positions were done using the PyroMark PCR kit (Qiagen) with 10 ng of converted DNA (except for HPV18‐L2 PCR, for which 20 ng of DNA was used) in a 25 µl volume with final concentration of reagents of 1× for Coral Load and PyroMark mix, 0.2 μM of PCR primers. PCR cycling conditions were 15 min at 94°C, followed by 45 cycles of 94°C, 54°C (51°C for HPV16‐L2, 55°C for HPV16‐URR), 72°C each for 30 sec and a final extension at 72°C for 10 min. PCR products were pyrosequenced using a PyroMark Q96 ID (Qiagen) instrument as previously described.^35^ Pyrosequencing runs included positive controls of known methylation level (0%, 50%, and 100%) to allow standardized direct comparisons between different primer sets and a negative control. For each gene and viral region, the methylation percentage was averaged over all the CpG positions investigated since we have previously shown that these CpGs are always similarly methylated within a particular sample.^21^, ^22^

For the main study on the LEEP sections, based on the pilot results obtained from the punch biopsies, we decided to concentrate on human gene *EPB41L3*, the L1 and L2 regions of HPV16 and the L2 region of HPV18 (Fig. 2). To the assays above, we also added newly developed assays for HPV31 and HPV33.^36^, ^37^ These were two CpG positions in the L1 region of HPV31 (6352 and 6354) and four positions in the L2 region of HPV33 (5557, 5560, 5566 and 5572). Assays were run as previously described.^22^ We used 8 µl of bisulfite converted DNA in the PCR using the PyroMark PCR kit as described above. Percentage methylation was averaged over all the CpG positions investigated and methylation levels were further averaged over all HPV types to create a single variable for HPV methylation called HPVme‐All (Fig. 2) because there were insufficient data for HPV18, HPV31, and HPV33 separately. For women with multiple samples of the same type (for example, when two CIN1 or three CIN3 areas were dissected), the percentage methylation was averaged to produce a single value per lesion type per woman (Fig. 2).

Assays were performed blinded by the technicians with cases and controls randomly intermixed, thereby minimizing concerns of biasing batch effects. We used a pre‐specified study design and the statistical analyses were done after molecular testing was finished and were independent of the team that produced the laboratory results.

### Statistical analysis

Analyses were performed according to a pre‐specified statistical analysis plan, which was blinded to the methylation data. For the pilot study using punch biopsies, Kruskal‐Wallis rank sum tests were used to compare averaged percentage methylation data between normal, CIN1 and CIN3 cases.

For the main study using LEEP samples, paired and unpaired Wilcoxon tests were performed as appropriate to compare DNA methylation levels between groups using *EPB41L3* and HPVme‐All as predictors. DNA methylation missing values of HPVme‐All were imputed with the value of zero for any hrHPV negative sample and by a median regression with age as a predictor and DNA methylation as an outcome for hrHPV positive samples. Missing values for *EPB41L3* were imputed by a median regression with age as a predictor and DNA methylation as an outcome independently of their HPV infection status (Fig. 2).

All *p*‐values were two‐sided with significance set at α < 0.05. No adjustments were made for multiple comparisons. Analyses were undertaken using R statistical software version 3.3.1.

## Results

### Pilot and main study: Specimens and strategy

A pilot study was performed on 104 punch biopsies to select a set of informative biomarkers for the main study. The methylation of candidate biomarkers *EPB41L3, MAL*, HPV16‐L1, HPV16‐L2, HPV16‐E2 binding sites (1, 3, and 4), and HPV18‐L2 is presented in Supporting Information Figure S2 and Table S1. *MAL* and the HPV16‐E2 binding sites were dropped from further study due to inadequate effect size. Although the L2 region of HPV18 was not significant in the pilot study due to small sample size (Supporting Information Fig. S2), methylation of this region was assayed in the main study because of the importance of HPV18 as a carcinogenic HPV. To these assays we added biomarkers in the L1 region of HPV31 and the L2 region of HPV33, which were validated as informative in different studies.^22^, ^36^

For the main study we used dissected LEEP tissues from 127 women (Fig. 2). The women were subdivided into CIN1 and CIN3 cases depending on the highest grade intraepithelial lesion diagnosed in the available tissues. There were 49 women classed as principal‐CIN1 (where no CIN3 was present on the cervix) and 78 as CIN3 cases. A majority (*n* = 53) of the CIN3 cases were classed as multifocal because they presented adjacent lesions of different grades (adjacent‐CIN1 or adjacent‐CIN3). The process gave us a total of 354 dissected CIN or normal areas (Fig. 2).

### Nature of methylation patterns in multifocal CIN3

**Figure 3:**
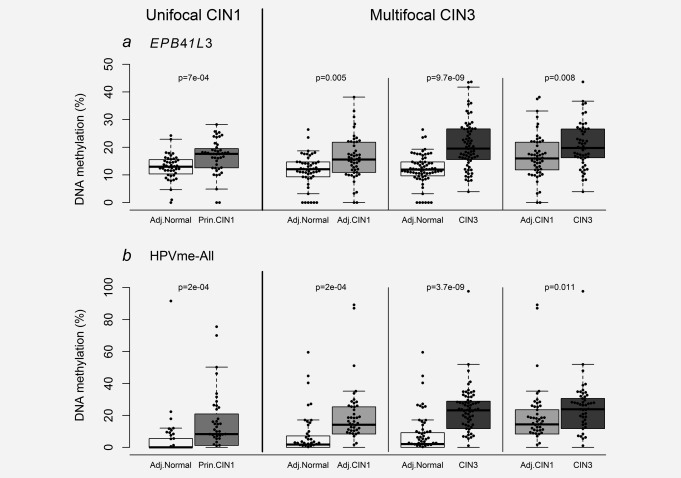
A paired analysis comparison of methylation levels between adjacent cervical tissue samples from the same women. Methylation levels of sample pairs from unifocal principal‐CIN1 cases are shown on the left panel and sample pairs from multifocal CIN3 cases on the right panel for human gene *EPB41L3* (*a*) and HPVme‐All (*b*). Adjacent normal tissue samples were significantly different from their concurrent CIN1 in both unifocal and multifocal cases (*EPB41L3* unifocal: *p* = 0.0007, and multifocal: *p* = 0.005; HPVme‐All unifocal: *p* = 0.0002 and multifocal cases: *p* = 2e‐04). In multifocal CIN3 cases, the CIN3 lesions were significantly different from the adjacent‐CIN1 lesions (*EPB41L3*: *p* = 0.008; HPVme‐All: *p* = 0.011). All pairwise comparisons were tested with Wilcoxon matched‐pairs signed rank tests. Abbreviations; Adj., adjacent; Prin., principal.

**Figure 4:**
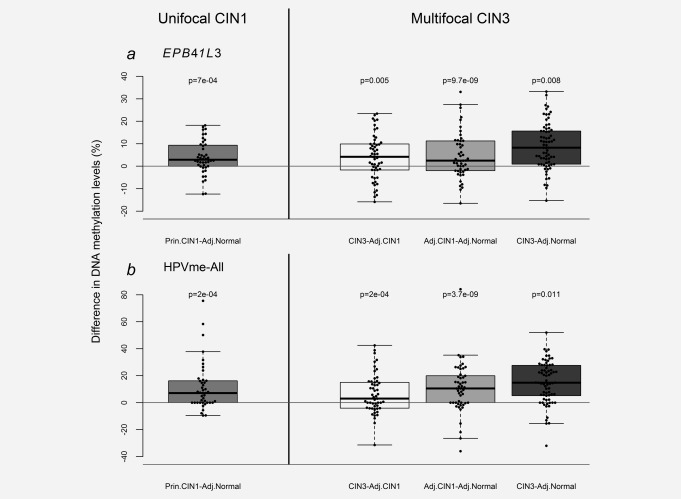
Differences in DNA methylation levels between adjacent lesions from the same cervix for *EPB41L3* (*a*) and HPVme‐All (*b*). The median differences were all positive and a large majority of individual differences were also positive, suggesting an increase of methylation with the severity of the lesion in cervical tissues of each woman. As expected the biggest difference was found between normal tissue samples and their adjacent CIN3 lesions in the multifocal CIN3 cases. Abbreviations; Adj., adjacent; Prin., principal.

A paired analysis on tissue isolated from the same cervices indicated that methylation of the normal tissue and CIN1 adjacent to CIN3 were significantly different from the CIN3 lesions. This was true for both *EPB41L3* (normal vs. CIN3: *p* < 0.0001 and CIN1 vs. CIN3: *p* = 0.008) and HPVme‐All (normal vs. CIN3: *p* < 0.0001 and CIN1 vs. CIN3: *p* = 0.0011; Fig. 3). The normal tissue samples were also different from the CIN1 lesions (*EPB41L3*: *p* = 0.005 and HPVme‐All: *p* = 0.0004). The average methylation of EPB41L3 increased with the disease progression. In the multifocal CIN3 cases, the trend was significant (Cuzick test for trend, *p* < 0.0001, χ^2^=38.9). Another way to view the data is to test the differences in methylation levels between the paired histopathological groups on a per‐cervix basis. In Figure 4, we plotted percentage differences in methylation between all paired measurements for *EPB41L3* and HPVme‐All markers. A large majority of methylation differences were positive, suggesting an increase of methylation as diagnoses changed from CIN1 to CIN3 in each woman.

### Normal tissue and CIN1 lesions have characteristic methylation levels regardless of whether they are from unifocal or multifocal lesional cervices

**Figure 5:**
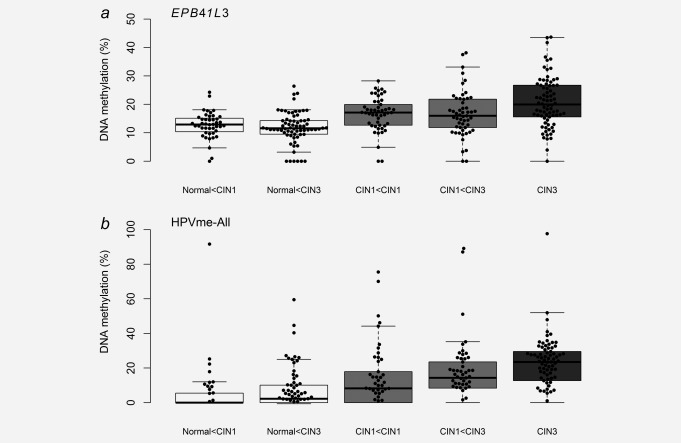
DNA methylation levels of normal (white), CIN1 (light gray) and CIN3 (dark gray) samples, analysed in an unpaired manner. Methylation levels of CIN1 specimens were similar whether originating from principal‐CIN1 cases (indicated by [CIN1 < CIN1] or from adjacent‐CIN1 near to CIN3 [CIN1 < CIN3] for both *a*) *EPB41L3* (*p* = 0.497) and (*b*) HPVme‐All (*p* = 0.110). Methylation patterns were also similar for normal tissues taken from CIN1 cases (normal < CIN1) and CIN3 cases (normal < CIN3) for *EPB41L3* (*p* = 0.212) and HPVme‐All (*p* = 0.163). All comparisons tested with Mann‐Whitney tests.

Methylation levels of normal tissues located near CIN1 or CIN3 were similar for both *EPB41L3* (*p* = 0.21) and HPVme‐All (*p* = 0.16; Fig. 5). Of greater interest, the pattern was the same for CIN1. Methylation levels of principal‐CIN1 were not different from adjacent‐CIN1 in CIN3 for *EPB41L3* (*p* = 0.49) nor HPVme‐All (*p* = 0.11; Fig. 5). In fact the median of *EPB41L3* for adjacent‐CIN1 appeared slightly (but non‐significantly) lower than for primary‐CIN1 and supports a model where *EPB41L3* DNA methylation levels may exist as discrete haplotypes, characterizing and possibly controlling the morphological appearance of pre‐cancerous lesions.

### Association of high‐risk HPV positivity and severity of lesions

**Figure 6:**
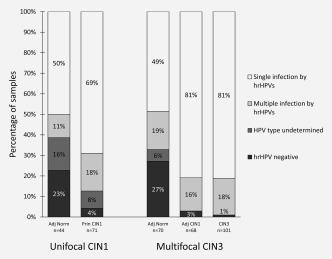
Percentage of samples infected by hrHPVs. In all sample types, single HPV infections were more frequent than multiple infections. Adjacent normal tissue samples presented a higher number of hrHPV negative samples (23% and 27% in unifocal principal‐CIN1 and multifocal CIN3 cases respectively) compared to the intraepithelial lesions: principal‐CIN1 (4%), adjacent‐CIN1 (3%) and CIN3 (1%). Most CIN1 lesions were infected by hrHPV types (87% of principal‐CIN1 and 97% in adjacent‐CIN1). All CIN3 samples, except one, were infected by hrHPV types. Abbreviations; Adj., adjacent; Norm, normal; Prin., principal.

354 dissected areas from LEEP samples were HPV typed (Fig. 6). A majority of principal‐CIN1 (87%), adjacent‐CIN1 (97%) and CIN3 (99%) were high‐risk HPV positive. Most of the CIN1 and CIN3 were infected by a single hrHPV type (69% of principal‐CIN1 and 81% adjacent‐CIN1). HPV infection was also found in normal tissues, but at a much lower rate (Fig. 6).

### Comparison of HPV types found in adjacent lesions and between LEEP samples and exfoliated cells

In multifocal CIN3, a majority of adjacent‐CIN1 were infected by the same hrHPV as the CIN3 (87%, Supporting Information Fig. S3). HPV type results were classed as compatible when some HPV types matched but one or the other paired sample also presented additional types. Normal tissues exhibited a somewhat lower similarity to their adjacent‐CIN1 (41% with same or compatible types to adjacent‐CIN1 and 45% compatible to principal‐CIN1) and adjacent CIN3 (51%). Furthermore, we noticed that when multiple lesions of the same grade were present in a multifocal CIN3, they were generally infected by the same HPV type (13/16 = 81% in CIN3 and 11/12 = 91% in CIN1), which is compatible with a single infection event from which multiple lesions arose. However, in principal‐CIN1 only 56% (9/16) of adjacent CIN1 were infected by the same HPV type, consistent with greater diversity of low‐grade lesions, perhaps due to multiple infection events combined with both transient and short‐term persistent HPV infections.

HPV types in macro‐dissected LEEP samples were also compared to their corresponding exfoliated cells from cervical scrapes at colposcopy and a good agreement was found between the two (Supporting Information Table S2). The HPV types found in 99% (77 out of 78) of the CIN3 and 88% (43 out of 49) of CIN1 lesions matched or were compatible (i.e., additional types were found either in cervical scrapes or in the dissected areas from LEEP) with the corresponding exfoliated cell genotyping results.

## Discussion

The main goal of our study was to characterize DNA methylation events associated with progression to CIN3. We show in our detailed methylation and HPV typing analyses that the topography of cervical HPV infection, the resultant intraepithelial neoplasia and the molecular patterns are heterogeneous and quite complex. We report here for the first time that, although adjacent CIN of different grades usually contain the same hrHPV type(s), methylation patterns are significantly different and characteristic of the lesion grade.

Previously published evidence of genetic alterations that accumulate during cervical tumorigenesis indicate a common cellular origin for multifocal lesions, suggesting that different intraepithelial lesions arise from the same or clonally related progenitor cells.^11^ The earlier study taken together with our results suggests that intraepithelial lesions of different grades on a multifocal cervix can arise simultaneously and therefore the “molecular switch” model (different methylation haplotype switches triggered at the same time) might be more likely than the “sequential progression” model (Fig. 1). If CIN3 predominantly arose directly from adjacent‐CIN1, we would expect to see methylation in the adjacent‐CIN1 having a bridging pattern. Methylation of the adjacent‐CIN1 should be very close to or at the same average level as the nearby CIN3 and such adjacent‐CIN1 should be distinctly different from primary‐CIN1; however, this was not what we observed. Furthermore, we would expect most CIN3 to have associated or adjacent‐CIN1, which was clearly not the case in our collection of samples. Although we cannot prove the actual overall secular mechanism of CIN3 origin from cross‐sectional data, our results do suggest a model where CIN3 can emerge directly from normal epithelium, possibly simultaneously to CIN1. After that, lesions that persist can enlarge over time but retain their characteristic epigenotypes. This model is also consistent with what we see with respect to HPV type concordance in different lesions on the same cervix. An initial rapid progression to CIN3 may perhaps be driven by a series of epigenetic changes happening both before and during HPV infection. We propose that distinct epigenotypes underpin distinct morphologic patterns such as CIN1 or CIN3, explaining partially the different progression characteristics of these lesions. The drivers of epigenetic changes could be multiple. One possibility is that epigenetic changes, such as *EPB41L3* methylation, may occur before hrHPV infection and facilitate the virus genome amplification and genetic instability phase, allowing in some women relatively rapid progression to CIN3. Furthermore, although most normal tissues have close to 0% methylation for *EPB41L3*, a sizeable proportion have modest elevation of *EPB41L3* methylation of up to 10%.^19^, ^24^

As regards to HPV types, we found that most CIN1 and CIN3 lesions (69% in unifocal CIN1 and 81% in multifocal CIN3 cases) were infected by a single hrHPV type. Our data partly confirm a similar study by Quint^32^ where an association of a single HPV type with a discrete area of CIN was found for 93% (372/399) of laser capture micro‐dissected tissue fragments they analysed by PCR. Van der Marel *et al*. have suggested that multiple high‐grade lesions on the cervix are often caused by a single carcinogenic genotype while other carcinogenic HPV types detected in cervical smears of the same patients are related to independent transient infections.^38^ They support the “one virus‐one lesion” hypothesis and discount biological interactions of multiple HPV infections on the lesion level.^32^

We also showed that when multiple lesions of the same grade were present in a multifocal CIN3, they were very often infected by the same HPV type (81% in CIN3 and 91% in CIN1). However, in principal‐CIN1 only 56% of the multiple CIN1 dissected were infected by the same HPV type. This indicates that when lower‐grade lesions are the primary diagnostic manifestation of HPV infection the distinct lesions are often infected by different and presumed transient HPV types.

There were consecutively large differences and an overall significant Cuzick test trend of increased methylation with increasingly severe intraepithelial histopathology, going from normal to CIN1 to CIN3. These effects were seen for both *EPB41L3* and HPVme‐All, and were very similar to results we obtained earlier on exfoliated cervical specimens. The correlation between biopsies and scrapes allows a generalization of our findings to specimens obtained by cervical scraping and vaginal self‐sampling.^24^, ^37^

Our study has some limitations. While the new model can underpin added insights for understanding epidemiologic and molecular distinctions between disease transitions in cervical pathogenesis a limitation is our inability to formally prove the methylation haplotype switch model. However, the same caveat applies to the sequential model, conception of which developed over decades, predominantly on the basis of logical but unproven assumptions. A definitive study would require lengthy (possibly decades long) follow‐up of large groups of women with HPV infection. The cervical lesions would require detailed topological mapping with frequent sampling of changing tissues areas by biopsies (which may affect the course of disease) and carefully directed scrapes, coupled to extensive molecular investigations. Such an *in vivo* mapping would no doubt quickly run into ethical concerns. We need to bear in mind that the two models are compatible and possibly both are correct in certain situations. Although our data support the idea that most CIN3 arise via a haplotype epigenetic switch some CIN3 may develop via a sequential progression.

A strength of our study is the use of an expertly rendered consensus CIN3 histopathological diagnosis as the primary endpoint rather than CIN2/3 or routine practice CIN3. Previous studies have shown that CIN2 is an equivocal diagnosis of precancer and more importantly in this context CIN2 has very poor inter‐rater agreement^27^, ^29^, ^39^, ^40^ and is much more likely to regress than CIN3.^41^, ^42^ Therefore, the grading of CIN2/3 may vary depending on the person rendering the diagnosis or on the follow‐up protocol, making comparisons of risks between studies including CIN2 as an endpoint challenging to interpret. In addition, we used a highly accurate pyrosequencing method of DNA methylation measurement, shown to have high precision and low bias, which provided quantitative results expressed as a percentage from 0 to 100. We performed a molecular examination of multifocal intraepithelial lesions within the same patients, with direct quantitative contrasts to adjacent lesions and lesions from other women.

While our data suggest at least two discrete epigenetic states (CIN1 and CIN3), we cannot say whether there are more epigenotypes. It is unlikely that our results were biased by the contamination of dissected lesions with other CIN or surrounding normal cells. All lesions were expertly marked by our team histopathologist (JC) and then dissected, carefully avoiding areas of other CIN and normal tissue. While the isolated CIN DNA probably had a low level of contamination from normal DNA the latter tissues were predominantly either not methylated or were methylated at lower levels than the CIN. Furthermore normal contamination on average would have been similar for principal‐CIN1 and adjacent‐CIN1, which were our most important comparison groups.

There are many remaining questions and it is clear that relatively little is known about specific genetic and epigenetic mutations that underpin the evolution of CIN3, nor exactly which steps have a required sequence that then further facilitate the transformation of CIN3 to malignancy. A lot is known about molecular and mechanistic aspects of oncogenes, tumor suppressor genes, regulatory RNA pathways, multi‐protein pathways etc. and also that many such pathways may become abnormal during carcinogenesis. However, for any given woman it remains not possible to predict accurately whether she will develop cervical cancer, what specific constellation of pathway abnormalities would lead to the cancer and over what timeframe this will occur.

In conclusion, we show for the first time that, although adjacent CIN of different grades usually contain the same hrHPV type(s), methylation patterns are significantly different and characteristic of the lesion grade, supporting a “molecular switch” model of progression to CIN3 that may be characterizable by distinct methylation haplotype patterns. Methylation testing may be an effective triage tool to detect and characterize women at high risk of developing CIN3 and cancer. Our results may influence the screening process in many ways, for example by providing an objective method to reach more accurate prognoses or by helping to avoid overtreatment of women with non‐progressive lesions. Reducing the numbers of younger women treated by surgical excisions would have an important effect in preserving the ability of women to have uncomplicated pregnancies and to overall improve childbearing at a later date.

## Financial Disclosure or Conflict of Interest

The authors declare no conflict of interest.

## Statement of Author Contributions

Study conception and design: C.R., A.L., and B.N.

Acquisition of data: C.R., S.B., J.C., R.B., and R.W.

Analysis and interpretation of data: B.N., C.R., A.L., A.A., and J.C.K.

Drafting of article: B.N., C.R., and A.L.

Critical revision: B.N., C.R., A.L., A.A., and J.C.K.

## Supporting information

Supporting Information Legends

Supporting Information Figure 1

Supporting Information Figure 2

Supporting Information Figure 3

Supporting Information Tables
