# Supplementary material for: Molecular progression to cervical precancer, epigenetic switch or sequential model?
Source: Int J Cancer. 2018 Jul 3;143(7):1720–30. doi: 10.1002/ijc.31549 (PMC6175180; doi:10.1002/ijc.31549)
Supplement: Supplementary file 1 — Supporting Information Legends [file IJC-143-1720-s001.docx]

**Supplementary Figures legends**

Supplementary figure 1. Methodology for sectioning, annotating and dissecting LEEP tissue blocks. Twelve 5um sections per block were cut. The first and last sections were used for haematoxylin and eosin staining (H&E) and sent for histopathological review and annotation. The annotated slides were used to dissect the CIN lesions and normal tissues of the remaining ten unstained sections. This material was used to purify DNA for subsequent analysis. Abbreviations; Adj: adjacent; Norm: normal; Prin: principal.

Supplementary figure 2. DNA methylation levels of normal, CIN1 and CIN3 punch biopsies for human genes *EPB41L3* (A) and *MAL* (B), HPV16 L1 region (C), L2 region (D), E2 binding site 1 (E), E2 binding sites 3 and 4 (F) and HPV 18 L2 region (G). Significance level is shown with the horizontal bars above the boxplots; KWT χ^2^: Kruskal-Wallis test statistic, CTT χ^2^: Cuzick test for trend statistic, ns: non-significant, *: p<0.05, **: p<0.001, ***: p<0.0001.

Supplementary figure 3**.** Comparison of HPV types found between adjacent tissue samples of different lesion grades. In the CIN3 cases, 87% of the adjacent-CIN1 lesions presented the same (or compatible) HPV type(s) as their nearby CIN3 lesions. A compatible type was defined as samples containing a matching HPV type in addition to other HPV types. Normal tissue samples showed fewer similarities when compared to adjacent-CIN1 (39%) or CIN3 samples (33%) in the multifocal CIN3 cases. This number amounted to 45% in principal-CIN1. A substantial number of normal samples were HPV negative and no comparison could be made for these samples. Abbreviations; Adj: adjacent; Norm: normal; Prin: principal.
