# Supplementary figures and images for: Molecular progression to cervical precancer, epigenetic switch or sequential model?
Source: Int J Cancer. 2018 Jul 3;143(7):1720–30. doi: 10.1002/ijc.31549 (PMC6175180; doi:10.1002/ijc.31549)

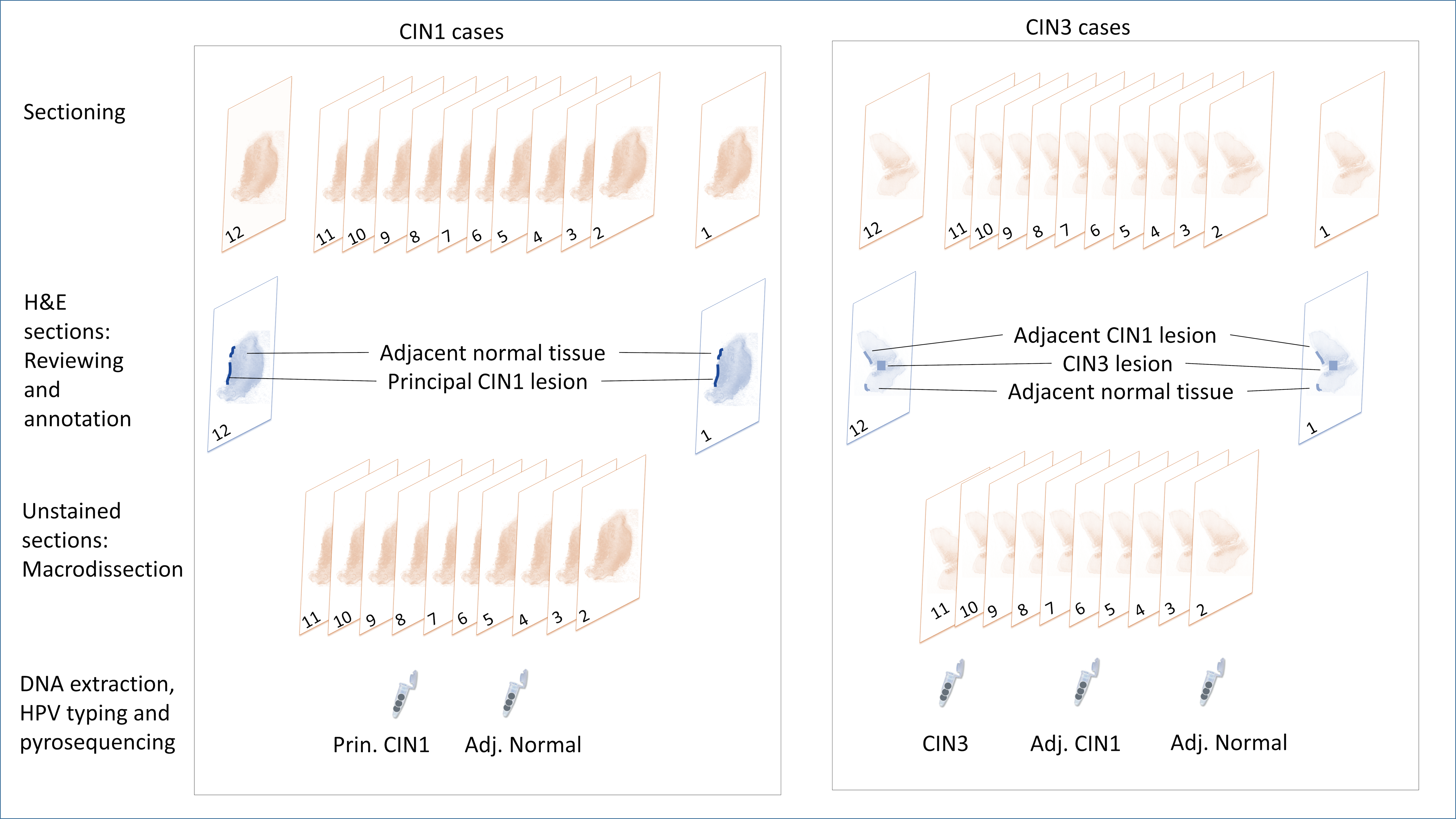

Supplement: Supplementary file 2 — Supporting Information Figure 1 [file IJC-143-1720-s002.tif]

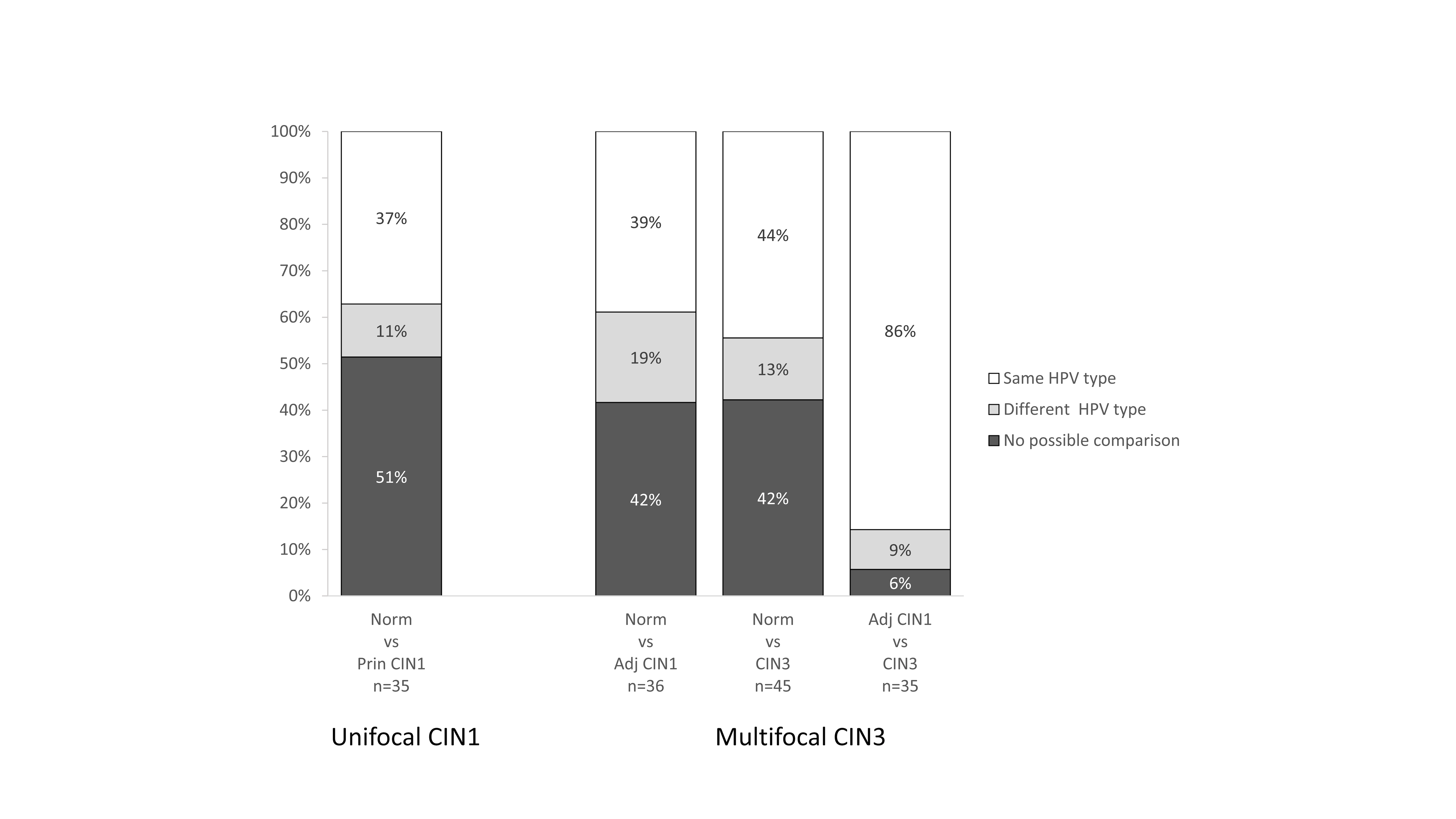

Supplement: Supplementary file 4 — Supporting Information Figure 3 [file IJC-143-1720-s004.tif]
