# Supplementary material for: Molecular progression to cervical precancer, epigenetic switch or sequential model?
Source: Int J Cancer. 2018 Jul 3;143(7):1720–30. doi: 10.1002/ijc.31549 (PMC6175180; doi:10.1002/ijc.31549)
Supplement: Supplementary file 5 — Supporting Information Tables [file IJC-143-1720-s005.docx]

**Tables**

Supplementary table 1. Methylation levels (%) of human genes and viral regions obtained in the pilot study for biopsies diagnosed as normal tissue, CIN1 and CIN3 lesions. n: number of samples with methylation results, KWT χ^2^: Kruskal-Wallis Test statistic, CTT χ^2^: Cuzick Test for Trend statistic.

| Gene/  HPV type | | HPV region | Normal median (n) | CIN1 median (n) | CIN3  median (n) | KWT χ^2^ | KWT  p-value | CTT  χ^2^ | CTT  p-value |
| --- | --- | --- | --- | --- | --- | --- | --- | --- | --- |
| *EPB41L3* | - | | 12.9 (15) | 14.2 (33) | 17.4 (56) | 9.99 | 0.0068 | 6.44 | 0.0110 |
| *MAL* | - | | 11.2 (14) | 10.4 (33) | 14.8 (56) | 4.42 | 0.1097 | 2.95 | 0.0860 |
| HPV16 | L1 | | 3.7 (9) | 15.3 (19) | 31.9 (35) | 19.19 | <0.0001 | 18.63 | <0.0001 |
| HPV16 | L2 | | 1.4 (7) | 10.7 (19) | 15.9 (30) | 22.02 | <0.0001 | 21.53 | <0.0001 |
| HPV16 | E2BS1 | | 5.48 (2) | 5.75 (15) | 8.8 (28) | 3.41 | 0.1820 | 2.68 | 0.1020 |
| HPV16 | E2BS3+4 | | 3.2 (2) | 0.76 (17) | 0.76 (31) | 3.14 | 0.2080 | 0.14 | 0.7100 |
| HPV18 | L2 | | 33.7 (2) | 26.5 (8) | 44.6 (13) | 0.66 | 0.7200 | 0.25 | 0.6200 |

Supplementary table 2. Comparison of HPV typing results between tissue samples macro-dissected from the LEEP and cervical scrapes obtained before colposcopy for CIN1 and CIN3 cases. A majority of the 49 CIN1 cases and 78 CIN3 cases showed infection with similar or compatible HPV types in their LEEP tissues and exfoliated cervical cells. A compatible HPV type was defined for samples that contained a matching HPV type in addition to other HPV types.

|  | CIN1 cases  n (%) | CIN3 cases  n (%) |
| --- | --- | --- |
| Same or compatible | 43 (88) | 77 (99) |
| Different | 1 (2) | 1 (1) |
| No possible comparison | 5 (10) | 0 |
| Grand total | 49 | 78 |
